# Disruption of Myelin Leads to Ectopic Expression of K_V_1.1 Channels with Abnormal Conductivity of Optic Nerve Axons in a Cuprizone-Induced Model of Demyelination

**DOI:** 10.1371/journal.pone.0087736

**Published:** 2014-02-03

**Authors:** Bandita Bagchi, Ahmed Al-Sabi, Seshu Kaza, Dimitri Scholz, Valerie B. O'Leary, J. Oliver Dolly, Saak V. Ovsepian

**Affiliations:** 1 International Centre for Neurotherapeutics, Dublin City University, Glasnevin, Dublin, Republic of Ireland; 2 Department of Biotechnology, Dublin City University, Glasnevin, Dublin, Republic of Ireland; 3 Conway Institute, University College Dublin, Belfield, Dublin, Ireland; 4 Deutsches Zentrum für Neurodegenerative Erkrankungen (DZNE), Ludwig-Maximilians-Universität München, Zentrum für Neuropathologie, Feodor-Lynen-Str. 23, Munich, Germany; Hospital Nacional de Parapléjicos - SESCAM, Spain

## Abstract

The molecular determinants of abnormal propagation of action potentials along axons and ectopic conductance in demyelinating diseases of the central nervous system, like multiple sclerosis (MS), are poorly defined. Widespread interruption of myelin occurs in several mouse models of demyelination, rendering them useful for research. Herein, considerable myelin loss is shown in the optic nerves of cuprizone-treated demyelinating mice. Immuno-fluorescence confocal analysis of the expression and distribution of voltage-activated K^+^ channels (K_V_1.1 and 1.2 α subunits) revealed their spread from typical juxta-paranodal (JXP) sites to nodes in demyelinated axons, albeit with a disproportionate increase in the level of K_V_1.1 subunit. Functionally, in contrast to monophasic compound action potentials (CAPs) recorded in controls, responses derived from optic nerves of cuprizone-treated mice displayed initial synchronous waveform followed by a dispersed component. Partial restoration of CAPs by broad spectrum (4-aminopyridine) or K_V_1.1-subunit selective (dendrotoxin K) blockers of K^+^ currents suggest enhanced K_V_1.1-mediated conductance in the demyelinated optic nerve. Biophysical profiling of K^+^ currents mediated by recombinant channels comprised of different K_V_1.1 and 1.2 stoichiometries revealed that the enrichment of K_V_1 channels K_V_1.1 subunit endows a decrease in the voltage threshold and accelerates the activation kinetics. Together with the morphometric data, these findings provide important clues to a molecular basis for temporal dispersion of CAPs and reduced excitability of demyelinated optic nerves, which could be of potential relevance to the patho-physiology of MS and related disorders.

## Introduction

Multiple sclerosis (MS) is a polyfactorial, devastating disease of the central nervous system (CNS). Despite being recognized almost two centuries ago, it remains the number one cause of non-traumatic neurological conditions in young adults [1], [2], with no radical treatment available. Throughout its protracted course, alternating deficits of axonal functions associated with demyelination deteriorates into conduction failure and progressive axonal degeneration, culminating in partial or complete sensory and motor incapacitation.

Functional and developmental studies have indicated essential roles for myelin in the rapid conduction of action potentials along thick myelinated axons [3], [4]. Enveloping neurites in a highly compartmented manner, myelin provides an effective shield essential for saltatory propagation of action potentials. There is considerable but conflicting evidence suggesting a stabilizing influence of voltage-activated K_V_1 currents on the excitability and conductivity of central and peripheral axons [5], [6], [7]. Mediated through channels produced by tetramerization of K_V_1.1 with 1.2 (and to a lesser extent 1.6) α subunits, and normally concentrated at the juxta-paranodes (JXPs), K_V_1 channels spread to internodes and nodal segments upon demyelination, causing impedance mismatch and disruption of action potential conduction [8], [9], [10]. Accordingly, indiscriminate pharmacological inhibition of K^+^ currents has been shown to restore the electrogenic functions of demyelinated axons, a mechanism that is implicated in some of the ameliorative influence of 4-aminopyridine (4-AP) and its analogues in MS patients [11], [12]. However, emerging evidence from animal studies suggests that the beneficial effects of therapeutically-relevant concentrations of 4-AP on axonal physiology are due to its action as a synaptic transmission enhancer [12], [13]. Indeed, low mM concentrations of 4-AP and 3,4-di-aminopyridine greatly facilitate neurotransmission at both excitatory and inhibitory synapses in the central and peripheral nervous systems [14], [15]. Of note, several studies also assigned therapeutic effects of 4-AP to its inhibition of immune cell proliferation [12], [16]. Inevitably, such broad-spectrum effects hampers the utilisation of 4-AP for discriminatory restoration of the functionality of demyelinated axons without off target effects.

A prevalence of optic neuropathies with functional disruptions during early MS [17], [18] kindled our interest in analysing the importance of K_V_1 currents in regulating electrophysiological properties of the optic nerve (ON) in a cuprizone-induced model of demyelination [19]. Our data demonstrate that demyelination is associated with an increase in K^+^ conductance mediated by ectopically expressed K_V_1 channels enriched with the K_V_1.1 α subunit. Evidence is presented for disruptive effects of K_V_1.1 on ON electrophysiology, and its critical influence on the biophysical and pharmacological profiles of K_V_1 currents in a heterologous expression system, signifying the pertinence of Kv1.1 subunit to conductive aberrations in demyelinating axons.

## Materials and Methods

### Animals and Induction of Demyelination

C57BL/6J male mice (8 weeks old) were obtained from Harlan (UK) and housed (21±2 °C, humidity 36±2% at 12/12-h light/dark cycle) in the Bio-Resource Unit of Dublin City University with food and water provided *ad libitum*. All procedures were approved by the University Ethics Committee, and licensed by the Department of Children and Health (Rep. of Ireland) in accordance with European Communities Council Directive of 24 November 1986 (86/609/ECC). Special efforts were made to minimize animal suffering and reduce the number of animals used. All animals received a Modified LabDiet®, with 0.2% cuprizone (Sigma, MO) supplemented to chow of the experimental (cuprizone treated) mice for 8 weeks, a time sufficient for induction of demyelination [19].

### Measurement of the Myelin Content in Brain Samples

Myelin was purified by sucrose density gradient centrifugation [20]. In brief, after decapitation of anaesthetized mice (sodium pentobarbital 200 mg/kg, i.p.), brains were dissected out, weighed, frozen in liquid N_2_ and stored at -80 °C till used. The tissue was homogenized in 0.25 M sucrose/phosphate buffer saline (PBS, pH 7.4) and centrifuged for 10 min at 500× g, 4°C; after re-centrifugation of the supernatant (10 min at 10000× g), the resultant pellet was re-suspended in 10 volumes of the buffered 0.25 M sucrose. Following addition of an equal volume of 0.88 M sucrose and centrifugation (3 h at 100000× g), myelin was collected at the interface between the two sucrose layers. Then, it was re-suspended in 10 volumes of ice-cold de-ionized water and re-centrifuged (15000× g for 30 min, 4°C); this step was repeated 4 times to remove the residual sucrose, with the final pellet being dried and weighed.

### Histochemistry and Transmission Electron Microscopy

Control and experimental mice (16 weeks old) were anaesthetized as above, perfused intra-cardially with PBS (pH 7.4) followed by fixation with 4% para-formaldehyde (PFA; Sigma, Ireland) in PBS, as described [21]. The brains were post-fixed overnight in PFA (4°C), cryo-protected (30% sucrose in PBS, 4°C for 24 h) and sectioned in mid sagittal plain (30 µm) followed by staining with luxol fast blue (LFB) or cresyl violet (CV) (n = 3 in each group), using protocols specified elsewhere [22], [23]. Sections were mounted, air-dried, immersed in xylene, and coverslipped with DPX mounting medium (Sigma, Ireland) and imaged using light microscope (Axioscope, Zeiss, Germany) with a DP72 colour camera (Olympus). For densitometry, colour images were converted into black and white, followed by analysis using intensity macro on randomly-defined regions of interests (ROI) in specified areas (ImageJ, NIH, USA).

For electron microscopy, perfusion of the mice was carried out according to the protocol [24] but with 1.5% glutaraldehyde in 0.1 M phosphate buffer (pH 7.2). After careful removal from the cranial cavity, ON was kept overnight in this fixative solution (4°C), post-fixed by 1% OsO_4_ for 2 h, and dehydrated. The quality of fixation and gross morphology of ON were assessed with toluidine staining of cross-sections (15 µm) while the rest of the tissue was immersed in graded alcohol and embedded in Epon resin. Ultra-thin sections (2 µm) were cut and contrasted with uranyl acetate/lead citrate, before being imaged with an FEI Tecnai-12 electron microscope (Tecnai FEI, Nanoport, Oregon, USA). The ‘g’ ratio or myelination index was estimated as the ratio of inner axonal diameter (d) to the outer diameter (D) of the ON fibres (g = d/D).

### Immuno-cytochemistry and Confocal Microscopy

Fixed ON embedded in Tissue Tack was frozen, sliced longitudinally (10 µm) (Leica CM3050S, Germany) and permeabilized (0.1% Triton X-100 in PBS) for 6 h (21°C) followed by blocking for 2 h with 10% goat serum [GS in PBS containing 5% bovine serum albumin (BSA) and 0.1% Triton X-100]. For K_V_1.1/1.2 double-staining, the primary anti-K_V_1.2 antibody (mouse monoclonal; NeuroMab, USA) was applied at 1∶500 dilution for 24 h (4°C) in 2% GS, 5% BSA, 0.1% Triton X-100 in PBS. After rinsing in PBS (3×20 min), sections were incubated overnight (4°C) with goat anti-mouse Alexa-568 fluor-labelled secondary antibody (Invitrogen, 1∶500) followed by washes (3×20 min) and 12 h exposure to anti-K_V_1.1 (rabbit polyclonal; Alomone Lab., Jerusalem). After 3 rinses, sections were incubated for 12 h in goat anti-rabbit-Alexa-488 flour labelled secondary antibodies (Invitrogen, 1∶1500), washed extensively, mounted and covered with Vectashield (Vector Labs, UK) for microscopic analysis. Specificities of the immuno-staining procedures were verified in negative controls, with omission of the primary antibodies. For Na_V_/K_V_1.2 staining, longitudinal cryosections (10 µm) of ON were placed on superfrost slides, blocked for 3 h with 10% horse serum containing 0.1% Triton X-100 (in PBS). Both polyclonal Na_v_ and monoclonal K_v_1.2 antibodies at dilutions of 1∶50 and 1∶200, respectively, were added for 24 h at room temperature, followed by 3 rinses. Subsequently, the tissue was incubated with biotinylated anti-mouse and -rabbit antibodies (Vector lab, 1∶1000) sequentially for 45 min each and developed with its corresponding streptavidin tagged flourophore (1∶1000, Alexa-488 and -568-labeled, Invitrogen) for 45 min, washed and mounted with Vectasheild for fluorescence microscopy. Field micrographs were obtained (20× objective) using a laser scanning microscope in epifluorescence mode (pinhole wide open) (AxioObserver, Carl Zeiss; Germany), while high-magnification images of JXPs were acquired in confocal mode (pinhole = 0.5AU, 40× objective) for analysis. Argon and Helium/Neon lasers provided the 488 and 568 nm lines for excitation; emitted signals were sampled in a frame mode at spatial resolution of 30 nm per pixel with 1.5 µs dwell time. The mean fluorescence intensities, fluorescent areas and co-localization of labelled K_V_1.1 and 1.2 α subunits were quantified with ImageJ and Zen 2008 (Carl Zeiss, Germany).

### CAP Recordings from ON and Pharmacological Analysis

Mice (16–17 weeks old) were decapitated under deep anaesthesia (as above) and ON carefully transected in proximity to the sphenoid canal. Brain with attached ON was removed and immersed for 5 min in bubbled (95% O_2_, 5% CO_2_) ice-cold solution containing (in mM): sucrose, 75; NaCl, 85; KCl, 2.5; NaH_2_PO_4_, 1.25; NaHCO_3_, 25; CaCl_2_, 0.5; MgCl_2_ 4; glucose, 25, pH 7.3 and glued in the recording chamber attached to the stage of an upright Olympus BX51WI microscope with the ON facing upward. The sample was perfused continuously throughout the experiment with bubbled (95% O_2_, 5% CO_2_) artificial cerebrospinal fluid (aCSF) containing (in mM): NaCl, 125; KCl, 3; NaH_2_PO_4_, 1.25; NaHCO_3_, 25; CaCl_2_, 2; MgCl_2_, 2; glucose, 25; pH 7.3 at 33–35°C. The perinervium was carefully removed with the distal end of the ON drawn into a suction electrode for stimulation. Evoked CAP recordings were made with low-resistance glass pipettes (5–15 µm tip diameter, 0.2–1.2 MΩ) filled with aCSF, which was gently inserted into the ON at close proximity to the optical chiasm to record evoked CAPs (stim. 100 µs pulse; 1.0–1.5 mA/0.03 Hz). Efforts were made to keep relatively constant the distance between the stimulation and recording electrodes at 1.0–1.2 mm. Unlike the complex shape CAP recordings of ON (Allen et al., 2006) using a suction electrode at room temperature, under our settings the CAP with glass pipette from control mice revealed a simple waveform, attributing the differences in the shapes CAP to: (1) smaller pool of axons contributing to the CAPs recorded with glass pipette (2) shorter distance between the stimulation and recordings sites with less temporal dispersion of the action potentials from population of heterogeneous axons and (3) use of temperatures close to physiological in the present study. Analog signals were acquired in episodic mode, amplified (EPC-10 USB controlled by Patchmaster 2.20; HEKA Instruments) and filtered at 10 kHz before storage for off-line analysis (Clampfit 10.0; Molecular Devices, CA). For measurement of the CAP refractory period, paired stimuli of sub-maximal intensity (1/2 max amplitude) were applied at various inter-pulse intervals, and the peak amplitude ratio of the second vs. the first (A2/A1) response was plotted as a function of the inter-stimulus intervals. Peptide blockers were aliquoted in recording solution, stored at −20°C and added to the perfusion medium before use. Dendrotoxin K (DTX_K_) was purified in-house; tityustoxin-Kα (TsTX-Kα) was obtained from Peptide International (Kentucky, USA). Tetraethylammonium chloride (TEA) and 4-AP were purchased from Sigma (Wicklow, Ireland) and Lancaster Synthesis (Lancaster, UK), respectively.

### Heterologous Expression and Characterization of Concatenated K_V_1 Channels

K_V_1.1 or 1.2 αsubunit genes were concatenated and expressed as homo-tetramers K_V_(1.1)_4_ or K_V_(1.2)_4_ and hetero-tetramers [K_V_(1.1)_2_–(1.2)_2_, K_V_1.1-1.2-1.1-1.1]; the tandem linking of the genes used an inter-subunit linker [25] derived from the untranslated regions (UTR) of the *Xenopus* β-globin gene (GenBank® accession number J00978). The cDNAs were amplified using K_V_X sequence-specific primers, as described [26]. Correct positioning of the genes in all of the pIRES2-EGFP plasmid constructs was confirmed by restriction analysis and DNA sequencing. Constructs were expressed in HEK293 cells (American Type Cell Culture, VA, USA) and surface biotinylation was performed as reported [27]. Western blotting with mouse mAb for K_V_1.1 or 1.2 was followed by their visualization with a goat anti-mouse secondary antibody conjugated to horseradish peroxidase. Macroscopic currents were measured from these cells by whole-cell voltage clamp recordings (EPC10, HEKA Elektronik, Germany). Patch pipettes (in-bath resistance1.5–3.0 MΩ) were filled with an internal solution (in mM): 95 KF, 20 KCl, 1 CaCl_2_, 1 MgCl_2_, 11 EGTA, 10 HEPES, 2 Na_2_ATP (pH 7.2 with KOH). External medium contained (in mM): 135 NaCl, 5 KCl, 2 CaCl_2_, 2 MgCl_2_ and 5 HEPES (pH 7.4 with NaOH). The liquid junction potential was corrected and series resistance compensated (70–80%). Conductance-voltage relationships were taken from the averages of the steady-state currents (100 ms before the termination of 300 ms pulse stimuli) activated with voltages of −80 to +20 mV with 5 mV increments. The activation rates were assessed through fitting the rising phase of K_V_1 currents with a single exponential function. Data were analysed by Pulsefit (HEKA Electronik, Germany) and fitted/plotted using Igor Pro 6 (WaveMetrics, USA). The IC_50_ values for inhibitors were obtained using automated whole-cell voltage clamp system (QPatch 16, Sophion Bioscience, Ballerup, Denmark), as previously described [26], with Qplate pin-wholes having resistances 2–3 MΩ. Giga-seals were formed upon execution of a combined suction/voltage protocol; gradually increasing suction leads to the whole cell configuration. Blockers were applied, via a four-way pipetting robot, through integrated glass-coated microfluidic flow channels. Data analysis was performed using an integrated database (Oracle) within QPatch software (Sophion Bioscience, Ballerup, Denmark). Peptide toxins were diluted from frozen aqueous stocks into external recording solution containing 0.01% (w/v) BSA and their inhibitory effects determined by the Hill equation fit to 7 concentrations.

### Statistical Analysis

All the data are presented as means ± S.E.M. *U*-Mann-Whitney, non-paired and paired Student's *t*-test was applied for comparison, with P<0.05 considered statistically significant.

## Results

### Widespread Demyelination in Axon-rich Tracts of CNS Induced by Cuprizone

**Figure 1:**
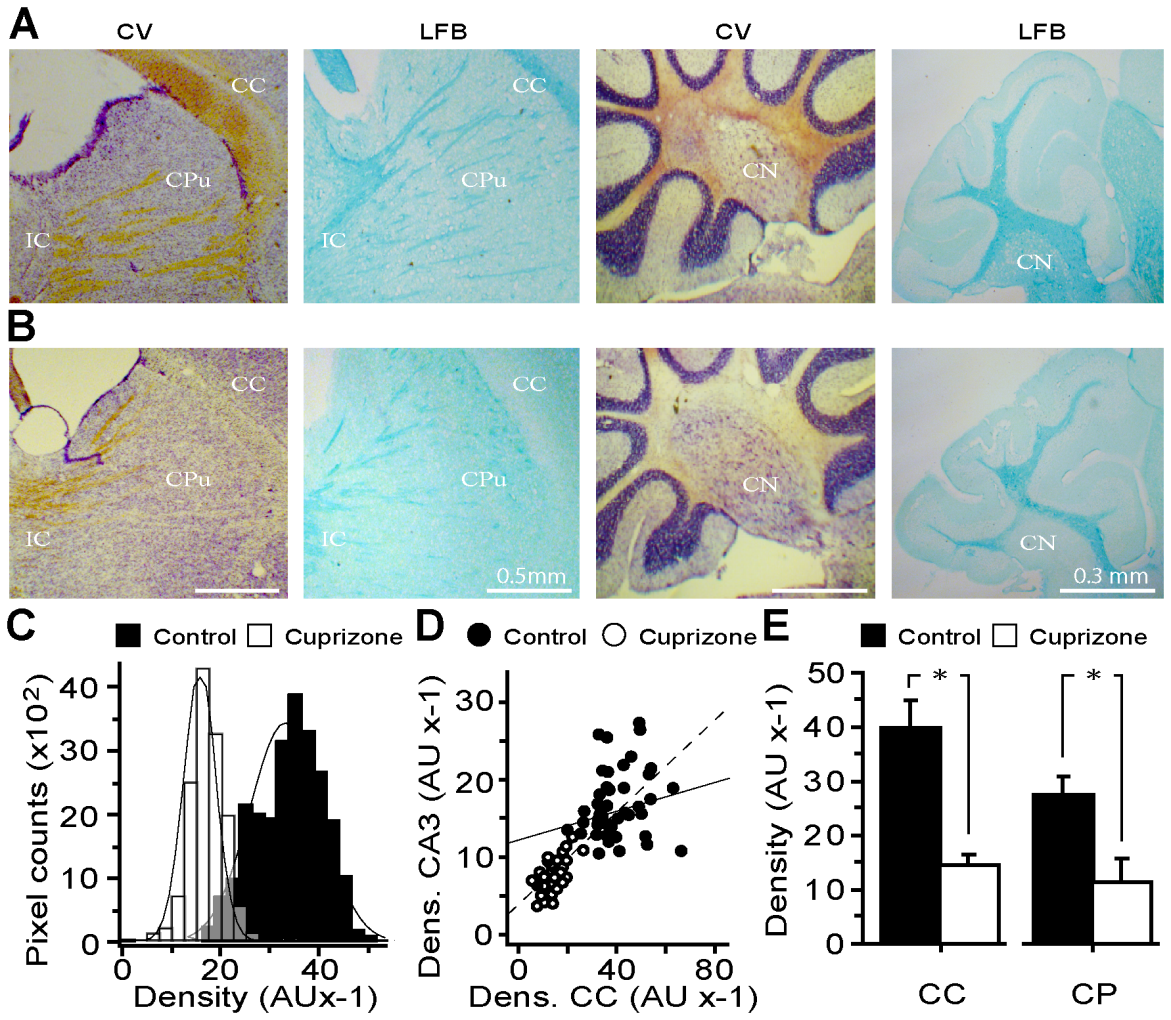
Cuprizone administration to mice induces widespread demyelination in several brain structures and reduces the myelin content. (**A–B**) Representative light micrographs of sagittal sections of brain from control and cuprizone-treated mice, respectively, stained for myelin with CV and LFB dyes. (**A**) Myelin is visualised in samples from the control mice, as light brown by CV and dense blue for LFB, in corpus callosum (CC), the stripes in the corpus striatum of the caudate putamen (CPu), internal capsule (IC) and the central nuclei of the cerebellar medulla (CN). (**B**) Extensive demyelination was observed in the above regions of treated mice. (**C**) Plot of the density distribution of myelin stained with LFB of defined colossal ROIs. A narrow and leftward-shift in the histogram of the density of myelin in cuprizone-treated samples reflects callosal demyelination (n = 6 in each group). (**D**) Correlation analysis of the density of LFB-stained myelin of random ROIs from callosal and hippocampal CA3 areas. Note the higher level of callosal myelin in controls (R^2^ = 0.21) compared to CA3 area, with its stronger decline in experimental samples (R^2^ = 0.54). (**E**) Summary histogram of the mean myelin density in CC and CP regions (>100 ROIs, from each group) with both areas revealing significant loss of myelin in mice that had received cuprizone. * p<0.05.

CNS demyelination was established in mice fed with cuprizone for 8 weeks. The animals did not show overt signs of ataxia, seizures, anorexia or other distress except that weight loss occurred during the first 2–3 weeks. Although this was followed by a gradual gain in weight by the end point, the treated mice remained underweight compared to controls (∼10%, n = 20, p<0.05). Analysis of brain sections stained for myelin with CV or LFB revealed its depletion especially notable in white matter-rich structures such as corpus callosum, internal capsule, stripes of the caudate nucleus and cerebellar peduncle (Fig. 1A, B). As evident from the histogram of myelin density in corpus callosum, a broader range of signals of higher intensity prevailed (36.7±0.37, ROI = 171) in controls compared to the values pulled from samples of cuprizone-treated mice that yielded more uniform signal densities of lower intensities (17.9±0.22, ROI = 146) (p<0.001; Fig. 1C). Cross-correlation analysis of relative myelin densities in callosal and hippocampal CA3 regions also unveiled prominent myelin loss in the hippocampus (Fig. 1D). Likewise, myelin loss was prominent in the cerebellar region of experimental group, with its substantial depletion in the paranuclear region of deep cerebellar nuclei and peduncular structures (Fig. 1A, right hand panels and E). These histochemical findings were corroborated by quantisation of myelin content of the brain tissue, which showed its significantly lower levels in total brain and forebrain of cuprizone-treated mice (56.2±7.4% and 71.3±8.7% of control, p<0.05; n = 3 in each group; not shown).

### Altered Compactness of Myelin and Axonal Geometry in ON of Cuprizone-treated Mice

**Figure 2:**
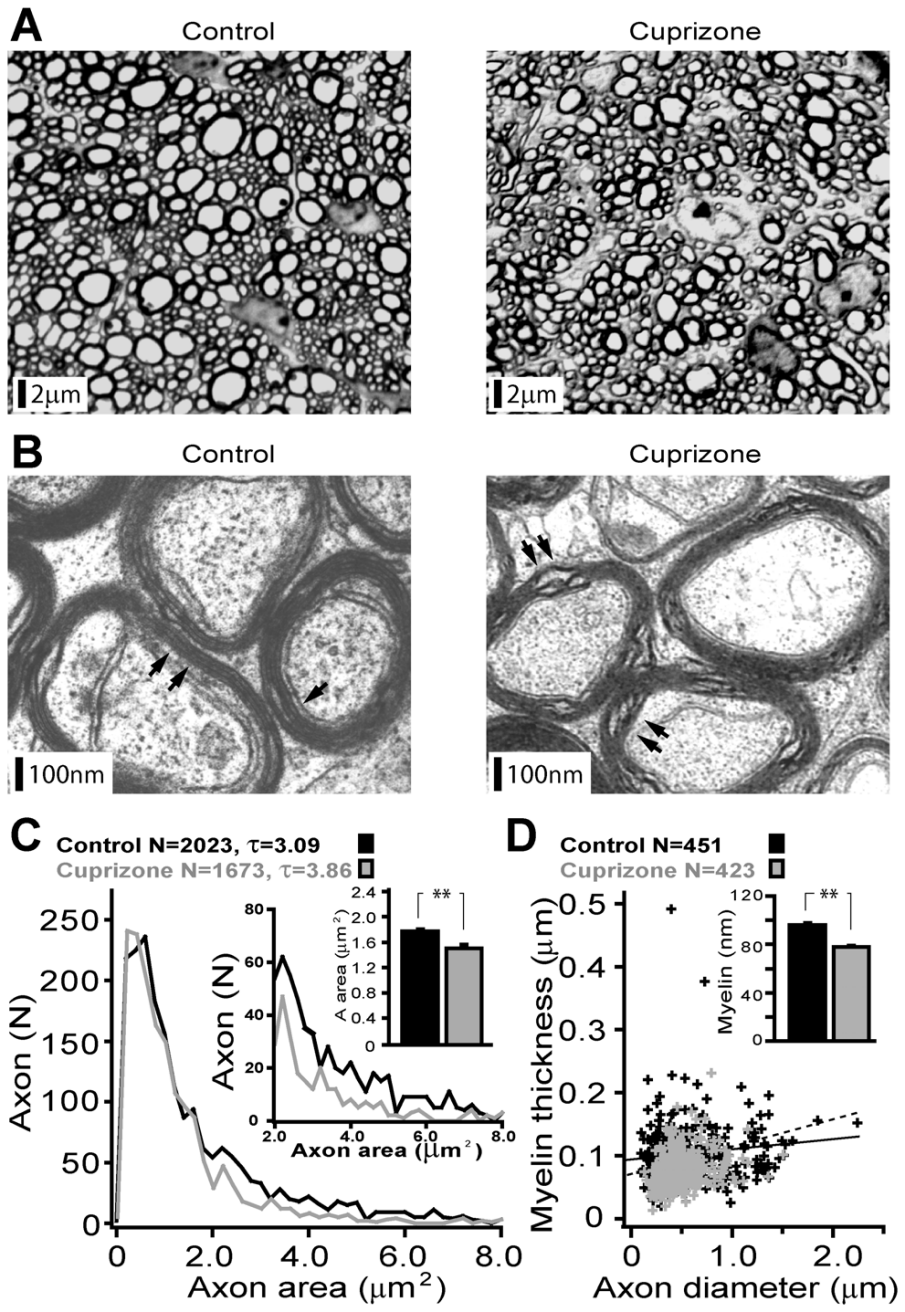
Light- and electron-microscopic analysis of ON reveals a decrease in the compactness and loss of myelin in the experimental mice. (**A**) Low power representative photomicrographs of ON from control and cuprizone-treated mice (TLB stained). Note less annular appearance of large diameter axons with a higher degree of intrinsic parcellation of the demyelinated nerve. (**B**) Electron micrographs of ON axons from control and treated mice. Along with a large number of myelinated axons (normal) with compact myelin sheaths consisting of several layers, axons covered with a loose myelin envelope of only a few lamellae were regularly encountered in the treated tissue (black arrows). (**C**) A summary plot of the distribution cross-sectional area of axons with insets highlighting divergence of this parameter for thicker axons (lower inset) and reduced mean cross-sectional area (upper inset) of axons in treated samples. (**D**) Graphical illustration of the relationship between myelin sheath thickness and axon diameter with regression lines and summary histogram of myelin thickness (inset) show a significant decrease in both parameters in treated mice.

Structural changes in ON axons from mice that received cuprizone relative to the controls were analyzed at light and electron microscopic levels. Figure 2A depicts low power micrographs of the toluidine blue stained cross-sections of ON. Axonal counts were comparable in two groups over the equivalent areas (15814±658 vs. 14069±955 over 50 µm^2^, p = 0.18). An absence of visible myelin breakdown and spheroid blebs accords with the lack of axonal degeneration in this model. Yet, individual axons of treated mice appeared less annular (Fig. 2A), with an overall reduced number of large calibre fibres (Fig. 2C, lower inset). Increased peri-axonal space with segregation of axons into irregular bundles along with occasionally visible reactive macrophage-like elements was also characteristic of the experimental samples (Fig. 2A). At the ultra-structural level, loosely myelinated axons with fewer lamellae were regularly encountered in the cuprizone-treated samples whereas the controls were typically enclosed in a compact and periodic sheath of myelin (Fig. 2B). Quantitative analysis of the axonal geometry and comparison with controls confirmed a notably lower fraction of large calibre axons (D = 0.474±0.02 µm vs. D = 0.437±0.01 µm; p<0.001) with an overall reduced axon cross-sectional area (Fig. 2C, upper inset). Estimates of the relationship between myelin thickness and axon diameter (Fig. 2D) unveiled a stronger correlation of these two parameters in ON from mice that were fed with cuprizone (R^2^ = 0.31 vs. R^2^ = 0.13, cuprizone-treated vs. controls), suggestive of a greater sensitivity of the myelin sheath thickness to cuprizone as compared to the axon diameter (Fig. 2D inset). Interestingly, the concurrent decrease of both parameters in axons maintains the estimated axonal ‘g’ ratio in treated samples relatively unaltered (n = 1019 vs. n = 910; control 0.845 vs. cuprizone 0.856, p = 0.17). Taken together, the evidence from light and electron microscopic data confirm substantial demyelination of the ON with changes in the morphometry of axons in the cuprizone treated mice, without any signs of their degeneration.

### K_V_1 Channel Distribution and Composition are Altered in the Demyelinated ON Axons

**Figure 3:**
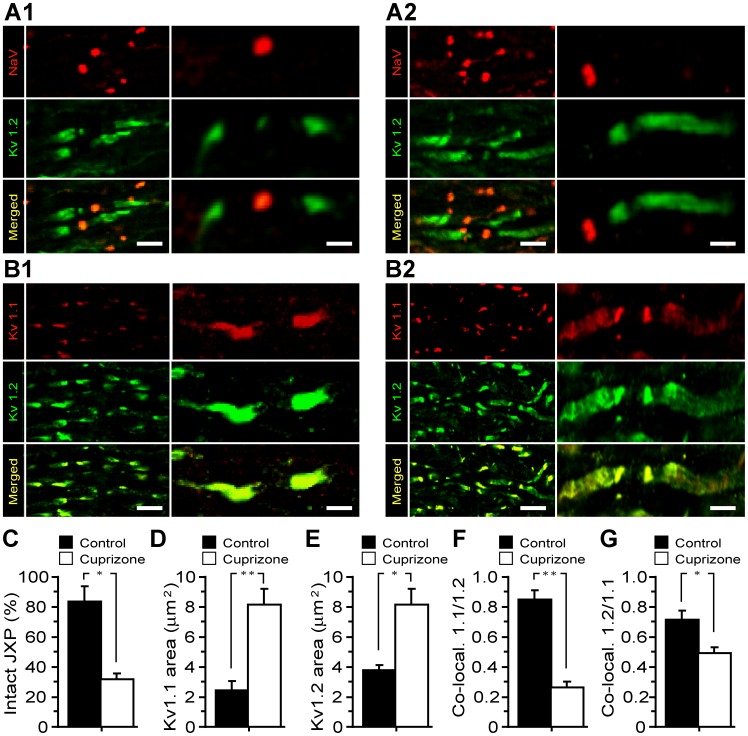
Demyelination alters the distribution and composition of K_V_1 channels in ON. Double [pan-Na (red)/K_V_1.2 (green)] immuno-labelling of control (**A1**) and experimental (**A2**) ON: note elongated JXPs with alterations in most of the nodal Na_V_ channel clusters in samples from the cuprizone-treated mice. (**B1–2**) Double immuno-labelling of ON for K_V_1.1 (red) and K_V_1.2 (green) subunits of K_V_1 channels: control (**B1**) and experimental (**B2**) samples, respectively. Note the highly localized occurrence of these proteins in JXPs of controls contrasting with their diffuse location along the ON axons in demyelinated specimens. Yellow staining corresponds to JXP regions showing co-localization of these proteins. The scale bars for low and high magnifications are 6 and 2 µm, respectively. (**C**) Summary histogram of the intact JXP labelled with anti-K_V_1.2 antibody of control and experimental ON axons (n = 3 in each group). (**D**) A plot of the mean area of JXPs labelled for K_V_1.1 channels in control (2.4±0.5 µm^2^) compared to the increased area of fluorescence intensity of JXPs in demyelinated (8.2±1 µm^2^) axons. (**E**) The mean fluorescence area of JXPs labelled for Kv1.2 channels in control (3.8±0.4) was lower than that in the treated ON axons (8.2±1 µm^2^). (**F**) A summary histogram of K_V_1.1 and 1.2 co-localization in control (0.86±0.06) and demyelinated (0.27±0.04) ON demonstrating a significant (p<0.001) reduction in the degree of K_V_1.1/1.2 co-localization in ON axons of the experimental mice. (**G**) The degree of K_V_1.2/1.1 co-localization in ON axons of the experimental mice showed a reduction, which is still significant (P<0.05), when comparing the control (0.71±0.06) and the demyelinated ON (0.49±0.04) values. Data are taken from control and demylinated ON axons of 3 animals, in each group.

In myelinated axons, there is a sharp segregation of voltage-dependent ion channels at the nodes of Ranvier with Na_V_ channels clustered within the nodal gaps while K_V_1 channels are located in the JXPs [28]. Both nodal and JXP regions were readily identified in control ON with pan-specific Na_V_ antibodies delineating nodes, whilst K_V_1.2 reactivity was most intense adjacent to the nodal gaps, decreased towards the internodes and gradually diminished to background level (Fig. 3A1). Visibly intact nodes were also detected in ON from the cuprizone-treated mice (Fig. 3A2), albeit representing only a fraction (33.3±2.2%) with the majority showing elongated K_V_1.2-positive JXPs (Fig. 3A2, C). In controls, double labelling with anti-K_V_1.1 and 1.2 specific antibodies revealed overlapping fluorescence, with both subunits flanking most of JXPs (Fig. 3B1). In contrast, the level of K_V_1.1 and 1.2 in the mice receiving cuprizone appeared increased (Fig. 3D, E), with K_V_1.1 subunit extending with greater prominence into the internodes (Fig. 3B2, D and E). These changes are readily reflected in high magnification confocal micrographs as elongation of K_V_1.1 and 1.2 labelled JXPs (3A2, B2, vs. 3A1, B1), in axons of cuprizone-treated mice and accord with their overall larger JXP areas (K_V_1.1∶2.4±0.5 µm^2^ vs. 8.2±1 µm^2^ p = 0.006; K_V_1.2∶3.8±0.4 vs. 8.2±1, p = 0.01) (Fig. 3D, E). Importantly, the co-localization coefficient of K_V_1.1/1.2 subunits within fluorescent profiles was higher in controls (K_V_1.1/1.2 = 0.86±0.06) compared to significantly diminished values in ON axons of cuprizone-treated mice (K_V_1.1/1.2 = 0.27±0.04), suggestive of a preferential increase in the expression level of K_V_1.1 in demyelinated axons (Fig. 3F compared to Fig. 3G). This inference is consistent with results of quantisation of relative luminescence-intensity (ELISA) and Western blot analysis which demonstrate considerable increase in the expression of K_V_1.1 subunits in ON of cuprizone-treated mice (Fig. S1) [29]. Finally, probing mouse ON (both Western blotting and immuno-fluorescence) failed to detect K_V_1.3, 1.5 and 1.6 subunits in ON axons from both controls and cuprizone-treated ONs except for a trace amount of K_V_1.4 (not shown).

### Altered Electro-responsiveness and Conductivity of ON from Cuprizone-treated Mice

**Figure 4:**
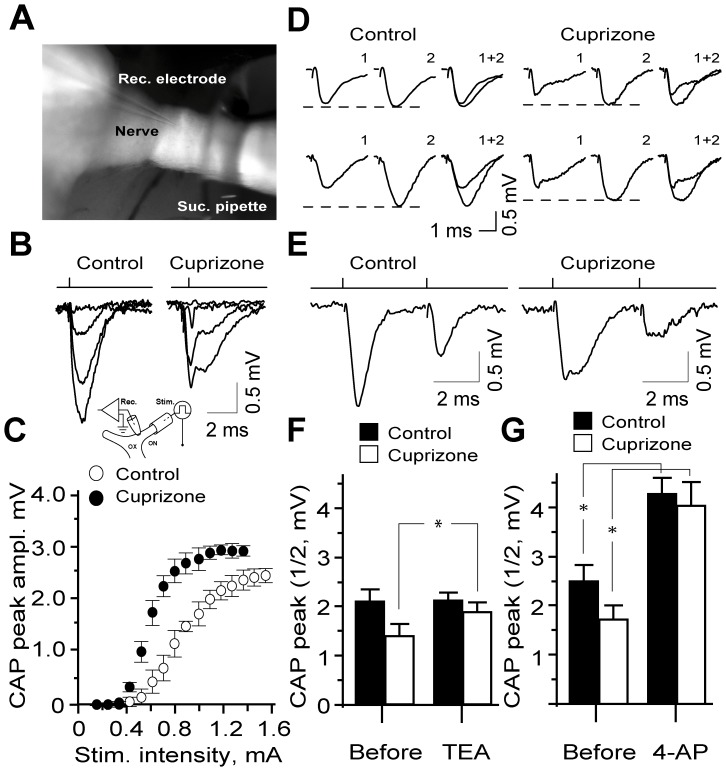
Demyelination disrupts the conductivity of ON axons which can be partially restored by 4-AP. (A, B) A low magnification micrograph (4×) demonstrating the semi-dissected ON (ventral view) with stimulation (suction, Suc. pipette) and recording (Rec.) electrodes. Graded synchronous CAPs recorded from control animals contrasting with bi-component CAPs derived from experimental ON activated from elevated stimuli thresholds (**C**). Insert illustrates the experimental set-up for CAPs recordings. Rec. - recording electrode; Suc. - suction pipette used for stimulation. ON – optic nerve, OX – optic xiasm. (**B**) Typical CAPs evoked in control ON by paired-pulse stimulation (PPS). Note the second CAP from the refractory phase following the first CAP. The evoked CAPs recorded from cuprizone-treated (demyelinated) ON axons showed lower amplitudes and protracted late components compared to the untreated (myelinated) ON axons. (**C**) Stimulus-response relation of CAPs in controls and experimental ON, showing lower activation threshold and higher amplitudes of evoked CAPs in demyelianted ON. (**D**) Representative recordings of CAPs from ON of control and cuprizone fed mice before (1) in the presence of TEA (2, upper row) or 4-AP (lower row) and (1+2) superimposed traces. (F, G) Summary of the effects of TEA (15–20 min application) on the CAPs in control and cuprizone-treated ONs (n = 5 in each group) (**E**) The summary histogram of CAP amplitudes scored before and after application of 1 mM 4-AP. Note the significant enhancement of the CAP amplitudes in demyelinated ON caused by 4- AP (P<0.05, n = 5 in each group).

CAP recordings were obtained from the proximal stump of semi-dissected ON at physiological temperature (Fig. 4A, B inset). In controls, stimulation of the distal end of the nerve evoked synchronous responses, whose amplitude could be graded by varying the stimulus strength (Fig. 4B); the threshold intensity for CAPs ranged between 0.36 and 0.44 mA (0.39±0.2; n = 6) and saturating at stimuli between 0.9 and 1.2 mA (0.96±0.4, n = 6) (Fig. 4C). Uniform conduction of ON axons in controls was evident from synchronous monophasic CAPs evoked by a single or paired pulse stimuli, with absolute refractory phases ranging between 2.0 and 2.6 ms (2.38±0.2, n = 6) (Fig. 4E). The potential reasons for discrepancies between the synchronous CAPs observed herein and those obtained using suction electrode (Allan et al., 2006) are given in MATERIALS AND METHODS. Analysis of the effects of TEA (10 mM) or 4-AP (1 mM) on sub-maximal CAPs of the intact ONs showed a significant increase in CAP amplitude by 4-AP, but not TEA after 15–20 min exposure (7.9%; n = 5; p>0.05 vs. 38.8%; n = 5; p<0.05) (Fig. 4D, F and G). Notably, neither TEA nor 4-AP affected the 50% refractory phase of CAPs (5.3±0.3 ms and 5.4±0.2 ms; p = 0.21 and p = 0.48) in controls, with only 4-AP reducing the threshold stimulus intensity (3.3±0.4; p = 0.038) for eliciting CAPs (not shown). Unlike the controls, CAPs of ON from cuprizone-treated mice revealed a distorted shape, with an early fast phase followed by a protracted late component (Fig. 4B, D and E), reflecting temporal dispersion of action potentials of axons in the demyelinated ON. Furthermore, the minimal stimulus intensity required for eliciting CAPs in the demyelinated sample was elevated (range: 0.42 and 0.62 mA, mean: 0.58±0.3; n = 5; Fig. 4C) with the absolute refractory phase prolonged (range: 2.8 and 3.6 ms; mean: 3.0±0.2, n = 5). In demyelinated nerves, blockade of K^+^ channels with TEA (10 mM) or 4-AP (1 mM) notably increased CAP amplitude (37.0%; n = 5; p<0.05 vs. 57.7%; n = 5; p<0.05), with the effects of the TEA also reaching statistical significance in controls (33.0%; n = 5; p<0.05) (Fig. 4D, F and G). As observed with the controls, refractory time for 50% recovery of CAPs (7.7±0.3 ms and 7.5±0.2 ms) remained unaltered under both treatments while the threshold stimulus intensity was significantly reduced (0.47±0.1 mA and 0.46±0.1 mA; TEA and 4-AP, respectively; n = 5 in each group; p<0.05).

### Differential Contribution of K_V_1.1 and K_V_1.2 to CAP in Demyelinated ON

**Figure 5:**
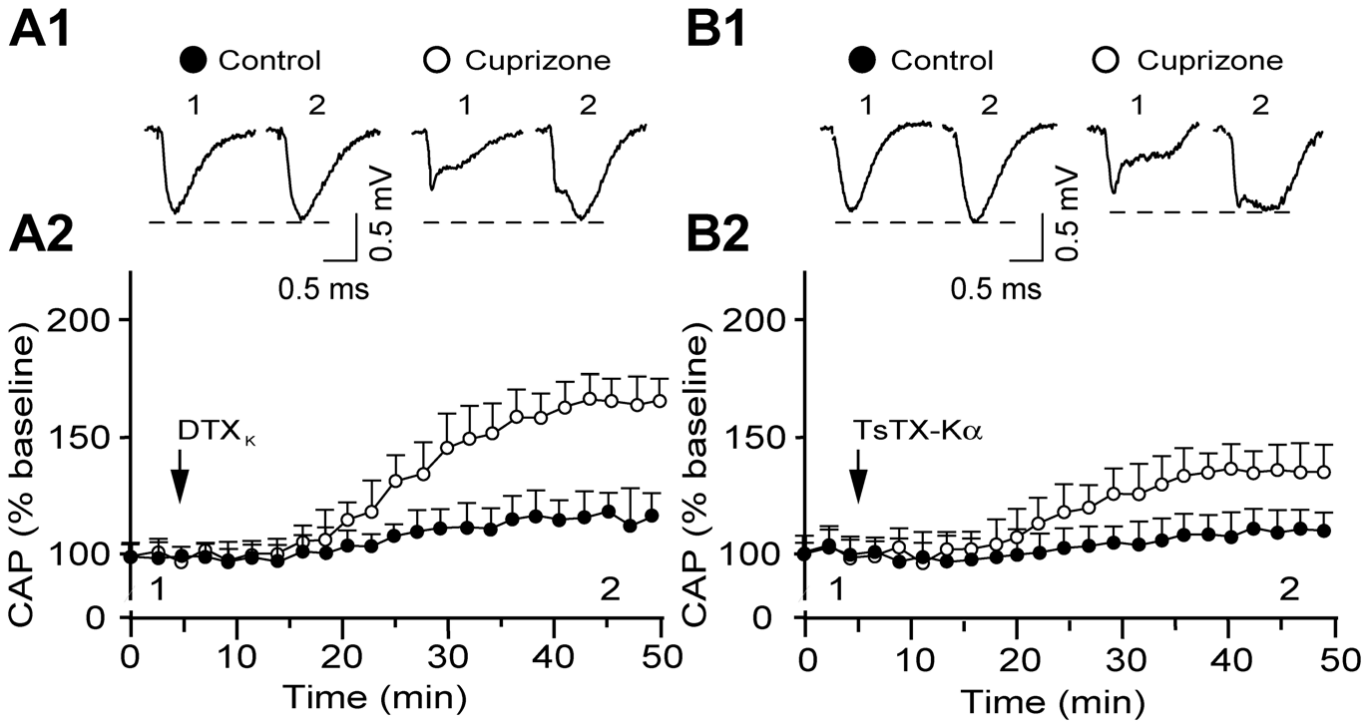
A more promenant contribution of K_V_1.1 than 1.2 subunits of K_v_ channels in regulating the excitability and conductivity of demyelinated ON. (**A1, B1**) Representative CAP recordings demonstrating the effects of DTX_K_ and TsTX-Kα respectively, before (1) and 40 min after presentation of the toxins to the control and experimental ONs (2). (**A2, B2**) Time course of the effects of DTX_K_ and TsTX-Kα, respectively, on evoked CAPs (sub-maximal) of control (filled circle; n = 5; n = 6) and experimental (open circle; n = 5; n = 5), respectively. Black arrows indicate the start of the application of toxins. Note a slight increase in the CAPs by these toxin blockers in controls (A1, B1, filled circle) compared to much stronger enhancement of CAPs in demyelinated ON by TsTX-Kα (B1 and B2) and especially DTX_K_ (A1 and A2).

Despite the presence of K_V_1.1 and 1.2 subunits in normal and demyelinated ON as demonstrated above, their relative contribution to tuning the electrogenic properties of axons therein remained elusive. Exposure of control ON to the potent blockers of K_V_1.1 and 1.2 subunits, DTX_K_ (100 nM) or TsTX-Kα (100 nM), respectively, caused no alterations in the characteristics of CAPs (Fig. 5A1, B1), with both amplitude and activation threshold remaining relatively unaltered (amplitude increase: 15±7.2%, n = 5; 10.3±5.8%, p>0.05; n = 6, respectively) (Fig. 5A2, B2). At the specified concentrations, DTX_K_ is known to block completely K_V_1.1-containing channels (IC_50_ of 2.5 nM) [30], [31] while TsTX-Kα abolishes K^+^ currents mediated by K_V_1.2 channels (IC_50_ of 0.55 nM) [32]. Because of the strong presence of both subunits in mouse ON JXPs (Fig. 3A1, B1), the ineffectiveness of such toxin blockers in intact axons is likely to be due to poor accessibility of the K^+^ channels. In stark contrast, in demyelinated nerves, the same concentrations of DTX_K_ and TsTX-Kα caused significant augmentation of the CAP amplitude (74.3±5.4%, n = 5; 32.2±4.2%, n = 5; respectively, p<0.05) with the late asynchronous component being particularly enhanced (Fig. 5A and B). Concurrently, both current thresholds (72.5% decrease with DTX_K_; 22.8% decrease with TsTX-Kα; P<0.05) and 50% refractory time were notably reduced, albeit the latter reached statistical significance only in DTX_K_ treated samples (61.3±7.1%, n = 5 vs. 13.2±3.1%, n = 6; p = 0.01 and p>0.05, respectively). The quantitatively different effects of these blockers was unexpected given that the presence of a single toxin-sensitive subunit renders hetero-tetrameric channels susceptible to toxins [33], and suggests that enhanced K^+^ conductance in demyelinated axons could be mediated largely, but not exclusively, through K_V_1.1 homo-tetrameric channels.

### K_V_1.1 Subunits Lower the Activation Threshold and Speed-up Activation Kinetics of K_V_1 Channels Recombinantly Expressed in Mammalian Cells

**Figure 6:**
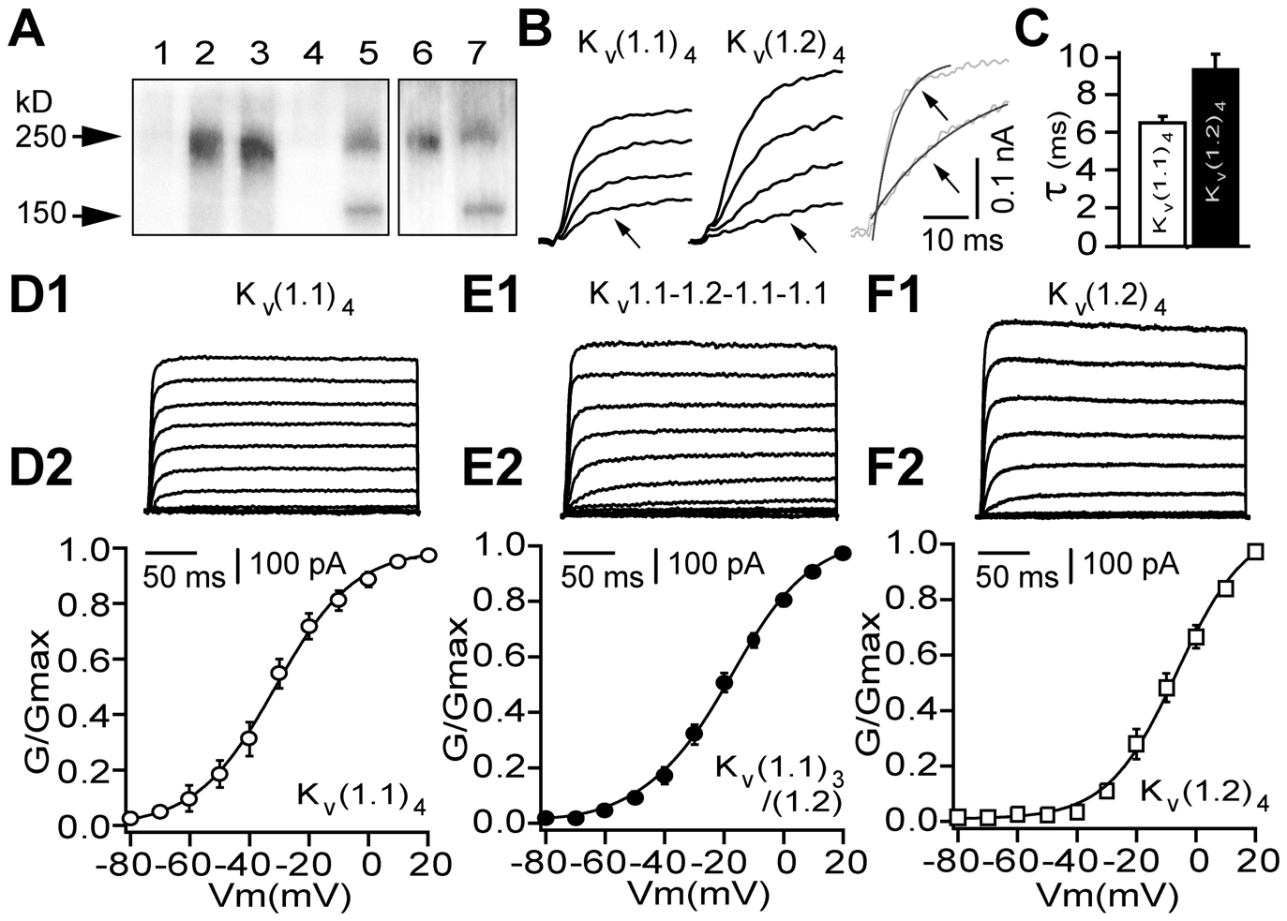
Functional characterization of recombinant K_V_1.1 homo-tetramers reveals distinctive biophysical profiles from those of K_V_1.1/1.2 heteromers. (**A**) Western blots of surface expressed concatenated K_V_1 channels in[HEK293 cells. Lanes: 1, non-transfected cells show no immuno-reactivity for K_V_1.1 (or K_V_1.2, not shown); 2, K_V_(1.1)_4_ and 3, K_V_1.1-1.1-1.2-1.1 detected with anti-K_V_1.1 IgG giving a band size of ∼250 kD; 4 and 6, K_V_(1.2)_4_ homo-tetramer was non-reactive with anti-K_V_1.1 IgG (4) but gave a distinct band when probed with K_V_1.2 IgG (6). Protein markers are indicated in lanes 5 and 7. (**B, D1–F1**) Representative recordings of macroscopic currents (300 ms pulse) from HEK293 cells transfected with the individual recombinant channels. (**B, C**) Activation rate of the voltage-dependent K^+^ currents mediated by K_V_(1.1)_4_ (left) and K_V_(1.2)_4_ (middle) channels (within the range of 10–30% of max. current) at 5 mV from indicated voltages (below) with super-imposed (right) representative traces from. A notable difference between the rates of activation of K_V_(1.1)_4_ and K_V_(1.2)_4_ is revealed by fitting the data with a single exponential (see **C**). (**D2–F2**) Conductance-voltage relations of macroscopic currents measured, based on the K^+^ current of the last 100 ms for each channel. Conductance at various command potentials were normalised and fitted with a single Boltzmann function. The difference in conductance values of K_V_(1.1)_4_ and K_V_(1.2)_4_ channel were statistically significant from −55 mV (P<0.05, Mann-Whitney *U*-test, see Table 1 for summary of the biophysical data).

To examine how demyelination-associated enrichment of K_V_1 channels with K_V_1.1 subunit could affect their functional properties, biophysical profiles of the currents mediated by concatenated homo-K_V_(1.1)_4_ or K_V_(1.2)_4_ and hetero-tetramers (K_V_1.1-1.2-1.1-1.1, K_V_1.1-1.1-1.2-1.2) were analysed. Expression of these tetramers in mammalian HEK293 cells was confirmed by surface biotinylation of the intact cells and Western blotting with anti-K_V_1.1 or 1.2 specific antibodies. This revealed a single band of the expected size (Mr ∼250 kD) for channels expressed on the plasmalemma (Fig. 6A), and their functionality was demonstrated by whole cell recordings. Each mediated voltage-activated non-inactivating K^+^ currents, which were consistently larger in cells expressing K_V_1.2 homo-tetramers or those containing this subunit together with K_V_1.1 (Fig. 6 D1-F1). Most importantly, K_V_1.1 homo-tetrameric channels activated at less depolarized thresholds than the currents resulting from the others (Fig. 5 D2-F2).This feature is reflected clearly in conductance-voltage (g_K_-V) plot of the K^+^ currents, with K_V_(1.1)_4_ activating from significantly more hyperpolarized potentials (close to -60 mV) compared to the K_V_1.1-1.2-1.1-1.1, K_V_1.1-1.1-1.2-1.2 and K_V_(1.2)_4_ channels (Fig. 6 D2-F2, Table 1). In all cells, the g_K_-V relationships of the K^+^ currents were fitted well with a Boltzmann function with half-maximal values of activation (V_1/2_) for K_V_(1.1)_4_ being most negative followed by intermediate potentials for the currents mediated by K_V_1.1-1.2-1.1-1.1 or K_V_1.1-1.1-1.2-1.2, and the most depolarised values observed with K_V_(1.2)_4_ channels (Table 1). Interestingly, significant differences were also observed between activation rates of these currents at near-threshold potentials, with K_V_(1.1)_4_ channel displaying a faster activation rate than the others (Fig. 6 B and C, D inset; Table 1).

**Table 1:** V_½_ for activation and onset rate of currents mediated by the different recombinant channels expressed in HEK293 cells.

Parameters	K_V_(1.1)_4_	K_V_1.1-1.2-1.1-1.1	K_V_1.1-1.2-1.1-1.1	K_V_(1.1)_4_
V_1/2_ (mV)	−35±1(6)	**−20±1(10)	#**−17±1(9)	**−7±1(8)
tau_1/2_ (ms)	13±2(6)	*18±1(8)	**24±1(8)	**29±2(8)

Results are represented as means ±S.E.M. (n-values);

* (p<0.05) and ** (p<0.005) numbers are significant compared to those from K_V_(1.1)_4_, (Mann Whitney *U*-test);

# data are taken from Al-Sabi et al., (2010).

### K_V_1.1- and K_V_1.2-containing Channels can be Distinguished by Selective Blockers

HEK293 cells expressing channels composed of homomeric K_V_(1.1)_4_, (1.2)_4_ or heteromeric combinations of both subunits [K_V_1.1-1.2-1.1-1.1 and [K_V_(1.1)_2_-K_V_(1.2)_2_] were used to mimic those possibly present in demyelinated ON axons. DTX_K_ potently and selectively inhibited only the K_V_1.1 homo-tetrameric channels, with sub-nanomolar IC_50_ (Table 2); introduction of a single K_V_1.2 subunit into the tetramer (K_V_1.1-1.2-1.1-1.1) lowered its susceptibility to blockade by DTX_K_. Having two copies of K_V_1.2 and K_V_1.1 subunits in the concatamer (K_V_1.1-1.1-1.2-1.2), resulted in an even lower sensitivity to DTX_K_ (IC_50_>100 nM). On the other hand, K_V_(1.2)_4_ channel was blocked by TsTX-Kα but apparently insensitive to DTX_K_ (Table 2). The K^+^ current elicited by a heteromeric channel with equal numbers of K_V_1.1 and 1.2 subunits proved ∼6 -fold less sensitive to TsTX-Kα than K_V_(1.2)_4_. Furthermore, TsTX-Kα failed to block K_V_1 channels containing 3 or 4 copies of K_V_1.1 subunits. Collectively, these results showed that DTX_K_ and TsTX-Kα are potent inhibitors of K^+^ currents mediated by K_V_1 channels that contain at least three copies of K_V_1.1 or two copies of 1.2 subunits, respectively.

**Table 2:** Differential inhibition of concatenated K_V_1 channels expressed in HEK293 cells by DTX_K_ and TsTX-Kα.

IC_50_ (nM)	DTX_K_	TsTX-Kα
K_V_(1.1)_4_	0.27±0.07 (5)	>100 (3)#
K_V_1.1-1.2-1.1-1.1	*4±0.1 (4)	>100 (3)
K_V_1.1-1.2-1.2-1.2	>100 (4)	15±2 (4)#
K_V_(1.2)_4_	>100 (3)	2.6±0.2 (3)#

Results are represented as means ±S.E.M.; n-values are in brackets;

* (p<0.05) numbers are significant compared to K_V_(1.1)_4_ (*t*-test),

# Data were taken from Al-Sabi et al., (2010).

## Discussion

The pervasive correlation between inflammatory optic neuropathies and symptoms of clinical MS, manifested by disruptions of visual functions, renders the ON an attractive experimental model. Being an anatomical extension of the forebrain [34], ON share key features of central myelinated tracts under healthy and disease conditions. Herein, a substantial demyelination with reduction of the myelin compactness and shrinkage of thick axons were demonstrated in ON from cuprizone-fed mice. Although the majority of nodes enriched with Na^+^ channels remained relatively intact, most of the JXPs became elongated due to spread and ectopic appearance of K^+^ channels composed of K_V_1.1 and 1.2 subunits in the inter-nodes, albeit with a disproportionate increase in the level of K_V_1.1. This inquisitive observation accords with the functional data from CAP recordings which highlighted better restoration of ON conductivity with DTX_K_-(K_V_1.1-selective) compared to TsTX-Kα (K_V_1.2-selective). Assessment of the K^+^ current mediated by recombinant (K_V_1.1)_4_ homo-tetrameric channels in HEK293 cells revealed a lower activation threshold and faster kinetics than those recorded for (K_V_1.2)_4_ homo-tetramers or K_V_1.2 subunit-containing hetero-tetramers. Thus, along with the demonstration of myelin loss and a decrease in the axon diameter, our data also provide important insights into demyelination-related changes in the molecular composition of K_V_1 channels in central axons, which could be of potential relevance to MS and other disease associated with the loss of myelin.

### ON Demyelination in Cuprizone-treated Mice: Relevance to MS

Models of virally- or chemically-induced (including by cuprizone) demyelination and experimental autoimmune-allergic encephalomyelitis have been widely used for studying de- and re-myelination processes in the CNS [35], [36]. High reproducibility with scattered lesions in the white matter, accompanied by edema and astrogliosis caused by cuprizone closely mimic changes occurring in the CNS during MS [37], [38]. Although some brain regions seem to be more sensitive to cuprizone than others, the widespread CNS demyelination involving corpus callosum, hippocampus, cerebellum, basal ganglia and other white matter rich brain structures have been documented [38], [39], [40]. In addition to reduced myelin content of the forebrain and cerebellum, we demonstrate for the first time pronounced myelin depletion of the ON in mice fed cuprizone. Indeed, electron microscopic analysis highlights reduction in the myelin thickness and compactness, along with shrinkage of the large diameter axons associated with the emergence of loose myelin stacks and sub-axolemmal vacuolar elements. A complete lack of myelin breakdown or axonal spheroid blebs in our experimental samples accord with an absence of degeneration of axons and neurons reported for this model [40]. Similar signs of axonopathy with reduction in the axonal caliber followed by degeneration at the later stages have been reported for transgenic mice lacking myelin proteins such as 2′,3′cyclic nucleotide 3′-phosphodiesterase, proteolipid protein and myelin-associated glycoprotein [41], [42] as well as in autopsies from MS brain [37]. These changes in axonal ultra-structure have been attributed to the fact that oligodendrocytes and myelin integrity, in addition to providing insulation, supply trophic support to axons which is essential for stability and normal functionality [43], [44]. Interestingly, the decrease in the myelin thickness of axons in our model was associated with moderate reduction of their diameter, perhaps a compensatory process, which retained the ‘g-ratio’ fairly normal. In fact, smaller axon diameter would reduce the capacitative load, favouring more effective propagation of action potentials through demyelinated segments; also, it would assist in maintaining ion homeostasis, delaying the onset of irreversible degeneration and neurological decline [45], [46]. It should be emphasized that even though the extent to which cuprizone demyelination reflects MS pathology in humans remains disputable [38], [39], extensive breakdown of myelin with its depletion in ON documented herein suggest this model as being useful and appropriate for exploring certain aspects of MS patho-biology.

### Functional Impact of Aberrant K_V_1 Channels in Demyelinated Axons

Even though clustering of Na^+^ channels in axons does not require the myelination process and developmentally precedes it, their lateral diffusion during demyelination suggests a stabilizing influence of axo-glial signalling on nodal Na^+^ channel clusters [47], [48]. In contrast, the myelin integrity appears to be mandatory for targeting K^+^ channels to JXPs [28], [49]. Accordingly, although antibodies to K_V_1.1 and 1.2 subunit labelled numerous JXPs in cuprizone-treated mice, many of these specializations were greatly elongated with K_V_1 proteins present in internodes, consistent with earlier documented dislocation of axonal K^+^ channels from their canonical sites in demyelination animal models and MS brain [49], [50]. Curiously, these changes in the distribution of K_V_1 channels appear to be associated with alterations of their molecular constituents, with co-localization analysis indicating disproportionate gain in K_V_1.1 immuno-reactivity with a notable advancement into the internodes of the demyelinated axons. In light of the established co-assembly of K_V_1.1 and 1.2 at JXPs throughout the brain [31], [49], preferential increase in the content of K_V_1.1 expression in the experimental mice suggests *de-novo* synthesis and post-translational enrichment of K_V_1 channels with this protein and, perhaps, expression of K_V_1.1 homo-tetramers. Notably, an asymmetric gain in the level of K_V_1.1 under demyelination has been demonstrated indirectly by *in situ* hybridization studies on *shiverer* mice. Affected by deletion of the 5′ exons of a gene encoding myelin basic protein, this model revealed much greater elevation in the level of K_V_1.1 transcripts (compared with K_V_1.2) [51]. Thus, along with stabilizing K_V_1 channels at JXPs, our data implicate an important role of axo-glial interactions in regulating their subunit composition. It is worth stressing that the occurrence of K_V_1.1 in the absence of other members of K_V_1 family has also been revealed in a minority of healthy peripheral axons but yet of unknown functionality [52].

Concurrently, electrophysiological data demonstrate partial restoration of CAPs and shortening of the refractive phase in demyelinated ON by K_V_1 channel blockers. Unlike CAPs in controls that are sensitive to 4-AP, in ON from mice treated with cuprizone, both TEA and 4-AP enhanced the population response. Nodal location of channels sensitive to 4-AP but not TEA may explain the discrepancy between the effects of these blockers. Such an interpretation is consistent with earlier studies, which demonstrated two pharmacologically-distinct K^+^ channel types, TEA and 4-AP-sensitive, in adult ON [53]. Because 4-AP (but not TEA) is able to diffuse through biological membranes [54], it could also act as an internal blocker of channels located in paranodal and internodal segments of healthy axons, another possible explanation of the differences between their effects. Interestingly, our data contrast with those from sciatic nerve where CAPs are insensitive to these blockers prior to and after their demyelination (Bostock et al., 1981). While improved conductivity of ON by 4-AP accords with its beneficial effects documented in MS clinical trials [12], [55], the broad spectrum of actions including pro-convulsive effects hinder its widespread use for MS therapy. Similar electrophysiological experiments with DTX_K_ and TsTX-Kα as K_V_1 channel blockers revealed the former be more effective in restoring CAPs in demyeliated axoms. It is worth stressing that in demyelinated samples these toxin blockers enhanced primarily the asynchronous late phase of CAPs, an effect attributable to their preferential action on slow conducting with compromised axon myelin sheath and enriched with ectopic K_V_1 channels accessible to these peptides. Because the relative strengths of K^+^ and Na^+^ currents in axons is a primary determinant of successful propagation of action potentials [9], the greater rescue of ON functions by DTX_K_ accords with enrichment of demyelinated axons with K_V_1.1-containing heteromers or homo-tetrameric K_V_1.1 channels.

### Implications for MS and other Demyelinating Disorders of the Central Nervous System

A well-established molecular mechanism for stabilizing the membrane potential of demyelinated axons is provided by Na^+^/K^+^ ATPase which, due to its electrogenic nature, provides a persistent hyperpolarizing drive during sustained activity, moving the axonal membrane potential away from the firing threshold [56]. An over-expression of K^+^ channels enriched with K_V_1.1 subunits in the ON axons from cuprizone-fed mice provides another, perhaps, equally powerful means for stabilizing the membrane potential at sub-threshold voltages. Unlike genetic knock-down of K_V_1.2 subunit (the main partner of K_V_1.1) associated with reduced excitability of central neurons [57], K_V_1.1 null mutants exhibit hyper-excitability and augmented axonal conductivity [6], [58], suggesting a powerful dampening influence of K_V_1.1-chonating channels on neuronal responsiveness. Indeed, the faster activation from more negative potentials of the K^+^ current mediated by K_v_1.1 subunit-dominated channels in HEK 293 cells could restrain and stabilize the axonal membrane at sub-threshold potentials. Considering the selective increase and ectopic expression of K_V_1.1 subunit in axons of demyelinated ON in relation to restoration of conductivity by DTX_K_ point to this being a potential target for ameliorative interventions. The sparse information available on specific molecular alterations responsible for impaired conductivity of demyelinated axons along with the poor selectivity of small K_V_ channel blockers with their considerable adverse effects have greatly hampered the development of effective restorative means. Interference of 4-AP, one of the most promising candidates, with remyelination and regeneration of impaired oligodendrocytes [59] renders its clinical use for rescuing axonal conductivity problematic; this stresses the urgent need for identification and evaluation of novel drug candidates. Hence, the recognition herein of novel K_V_1 channels enriched with K_V_1.1 subunit represent a significant step forward towards the development of a specific extra-cellular blocker of such channels with potential for recovering the conductivity of demyelinated axons. Nevertheless, research on human specimens is warranted to ascertain if the demyelination-associated changes in the composition of K_V_1 channels described herein is applies to central axons affected by MS.

## Supporting Information

Figure S1 (A, B) Quantification of the K_V_1.1 and K_V_1.2 α subunits in detergent-solubilized extracts of optic nerve, using Western blot (WB) analysis (A) and chemiluminisence ELISA (B). (A) Representative blots of K_V_1.1 and K_V_1.2 subunits of K_V_1 channel from mouse optic nerve. Mouse monoclonal IgGs for K_V_1.1 and 1.2 were used for detecting the protein (bands) of interest. The positions of markers shown on the left side indicate the molecular weight (kD). For Western blotting 15 µg crude membrane protein was loaded on each track of the gel. Note considerably denser K_V_1.1 band for material from cuprizone-treated mice. (B) ELISA based quantification of K_V_1.1 subunit protein shows significant increase of its level in an extract of optic nerve from cuprizone-treated mice (p<0.05; paired Student’s *t*-test). The signals were quantified as relative luminescence intensity; the relative values for untreated control and cuprizone-treated sample derived from the known standards are presented (for further details, Bagchi, 2013).
(DOC)
