# Supplementary material for: Disruption of Myelin Leads to Ectopic Expression of KV1.1 Channels with Abnormal Conductivity of Optic Nerve Axons in a Cuprizone-Induced Model of Demyelination
Source: PLoS One. 2014 Feb 3;9(2):e87736. doi: 10.1371/journal.pone.0087736 (PMC3912067; doi:10.1371/journal.pone.0087736)
Supplement: Figure S1 — (A, B) Quantification of the KV1.1 and KV1.2 α subunits in detergent-solubilized extracts of optic nerve, using Western blot (WB) analysis (A) and chemiluminisence ELISA (B). (A) Representative blots of KV1.1 and KV1.2 subunits of KV1 channel from mouse optic nerve. Mouse monoclonal IgGs for KV1.1 and 1.2 were used for detecting the protein (bands) of interest. The positions of markers shown on the left side indicate the molecular weight (kD). For Western blotting 15 µg crude membrane protein was loaded on each track of the gel. Note considerably denser KV1.1 band for material from cuprizone-treated mice. (B) ELISA based quantification of KV1.1 subunit protein shows significant increase of its level in an extract of optic nerve from cuprizone-treated mice (p<0.05; paired Student’s t-test). The signals were quantified as relative luminescence intensity; the relative values for untreated control and cuprizone-treated sample derived from the known standards are presented (for further details, Bagchi, 2013). (DOC) [file pone.0087736.s001.doc]

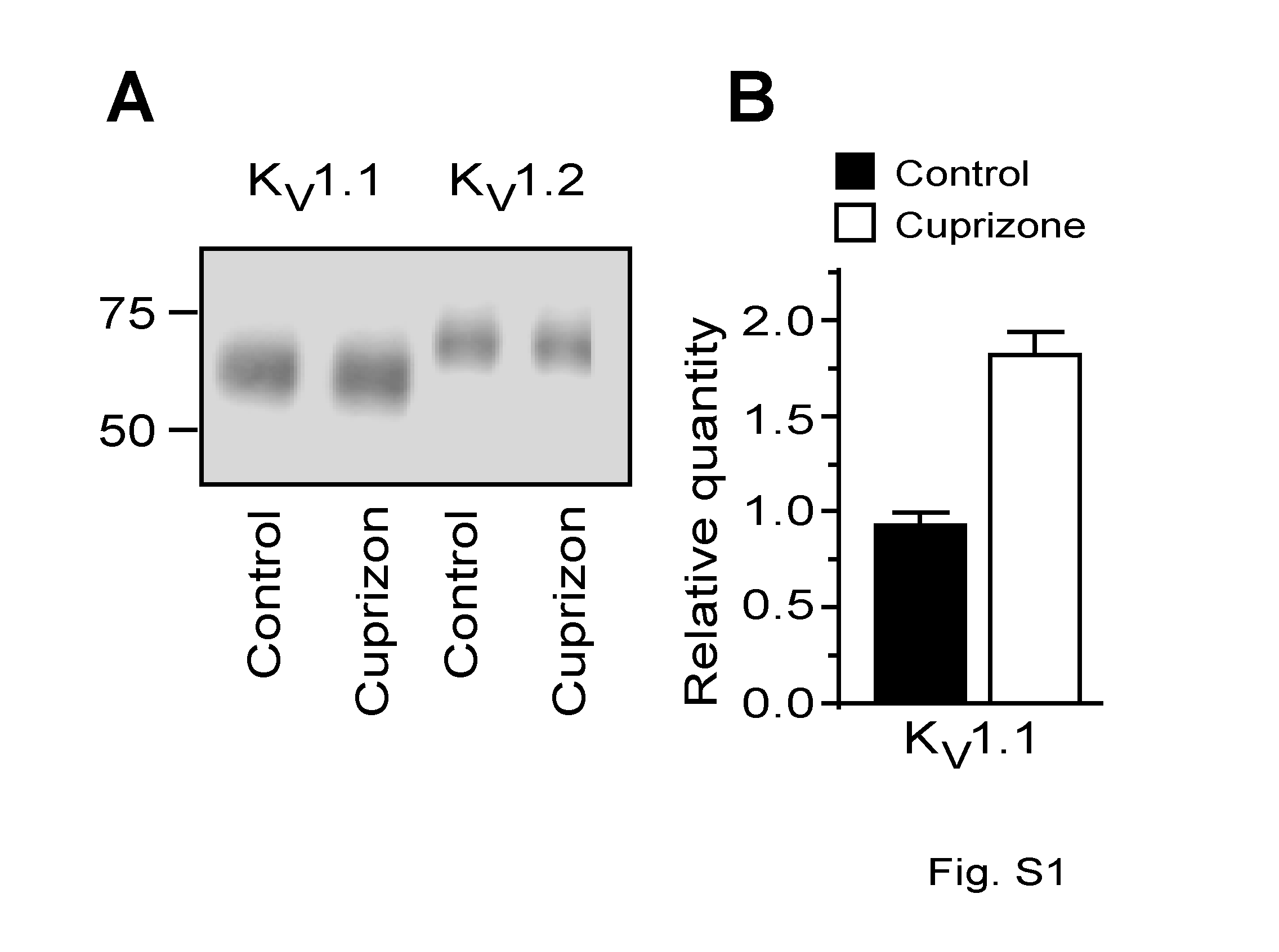


**SUPPLEMENTARY MATERIAL**

**Fig. S1:** (A, B) Quantification of the K_V_1.1 and K_V_1.2 α subunits in detergent-solubilized extracts of optic nerve, using Western blot (WB) analysis (A) and chemiluminisence ELISA (B). (A) Representative blots of K_V_1.1 and K_V_1.2 subunits of K_V_1 channel from mouse optic nerve. Mouse monoclonal IgGs for K_V_1.1 and 1.2 were used for detecting the protein (bands) of interest. The positions of markers shown on the left side indicate the molecular weight (kD). For Western blotting 15 μg crude membrane protein was loaded on each track of the gel. Note considerably denser K_V_1.1 band for material from cuprizone-treated mice. (B) ELISA based quantification of K_V_1.1 subunit protein shows significant increase of its level in an extract of optic nerve from cuprizone-treated mice (p < 0.05; paired Student’s *t*-test). The signals were quantified as relative luminescence intensity; the relative values for untreated control and cuprizone-treated sample derived from the known standards are presented (for further details, Bagchi, 2013).
